# Vermicompost application improves leaf physiological activity, 2-acetyl-1-pyrroline, and grain yield of fragrant rice through efficient nitrogen assimilation under Cd stress

**DOI:** 10.3389/fpls.2024.1481372

**Published:** 2024-12-02

**Authors:** Anas Iqbal, Xiaoyuan Chen, Rayyan Khan, Maid Zaman, Aamir Hamid Khan, Marcin Kiedrzyński, Mohamed Ebaid, Abdulwahed Fahad Alrefaei, Sobhi F. Lamlom, Xiangru Tang, Muhammad Zeeshan

**Affiliations:** ^1^ College of Agriculture, South China Agricultural University, Guangzhou, China; ^2^ Yingdong College of Biology and Agriculture, Shaoguan University, Shaoguan, China; ^3^ Key Laboratory of Crop Cultivation and Physiology, College of Agriculture, Guangxi University, Nanning, China; ^4^ Department of Entomology, University of Haripur, Khyber Pakhtunkhwa, Haripur, Pakistan; ^5^ Department of Biogeography, Paleoecology and Nature Conservation, Faculty of Biology and Environmental Protection, University of Lodz, Lodz, Poland; ^6^ Plant Production Department, Arid Lands Cultivation Research Institute (ALCRI), City of Scientific Research and Technological Applications (SRTA-City), New Borg El-Arab City, Alexandria, Egypt; ^7^ Department of Zoology, College of Science, King Saud University, Riyadh, Saudi Arabia; ^8^ Plant Production Department, Faculty of Agriculture Saba Basha, Alexandria University, Alexandria, Egypt

**Keywords:** vermicompost, cadmium toxicity, fragrant rice, 2-Acetyl-1-pyrroline, leaf physiological activity, N metabolism, metalloid stress

## Abstract

Cadmium (Cd) pollution in arable soils and its accumulation in rice plants have become a global concern because of their harmful effects on crop yield and human health. The *in-situ* stabilization method which involves the application of organic amendments such as vermicompost (VC), is frequently utilized for the remediation of Cd-contaminated soils. This study investigated the effects of VC on the soil chemical properties and the physio-biochemical functions of fragrant rice, as well as nitrogen (N) metabolism and assimilatory enzyme activities, 2-acetyl-1-pyrroline (2AP) content in rice grains, and the grain yields of fragrant rice cultivars, i.e., Xiangyaxiangzhan (XGZ) and.Meixiangzhan-2 (MXZ-2) under Cd stress condition. Four doses of VC (.VC1 = 0, VC2 = 3 t. ha^-1^, VC3 = 4 t ha^-1^, and VC4 = 6 t ha^-1^) and two levels of Cd (0 and 25 mg Cd kg^-1^) were used in this study. Our results showed that VC supplementation significantly (*p* < 0.05) improved soil characteristics, including soil organic carbon, available N, total N, phosphorus (P), and potassium (K). Furthermore, VC enhanced plant physiological and biochemical attributes in fragrant rice, such as net photosynthetic rate (*Pn*), nitrate reductase (NR), nitrite reductase (NiR), glutamine synthetase (GS), glutamate oxoglutarate aminotransferase (GOGAT) enzyme activities, protein contents, amino acid, and 2-acetyl-1-pyrroline (2AP) contents under Cd stress condition. Specifically, the VC-amended treatment, Cd2 + VC3, led to an 86.75% increase in *Pn* and 2AP, and a 60.05% and 77.55% increase in grain yield for MXZ-2 and XGZ cultivars, respectively, compared to Cd-only treated plants (Cd2 + VC1). In addition, VC application significantly (*p* < 0.05) decreased the Cd uptake and accumulation in rice plants. The correlation analysis indicated that leaf physiological activity and biochemical traits are strongly correlated with soil qualitative traits, suggesting that improved soil health leads to enhanced leaf physiological activity, N metabolism, grain 2AP content, and grain yields. Among the treatments, Cd2 + VC3 showed the best performance in terms of soil fertility and rice quality and production. Consequently, our study indicates that using VC in soils may benefit rice growers by improving soil fertility and supporting sustainable rice productivity and quality in soils contaminated with Cd.

## 1 Introduction

Fragrant rice is considered premium quality and is globally recognized for its distinct aroma and flavor (Bryant and McClung, 2011). Fragrant rice cultivars release unique aromatic components that set them apart from non-fragrant rice cultivars (Jezussek et al., 2002). Several volatile compounds have been identified in fragrant rice, such as 2AP, 4,5-epoxy-(E)-2-decennial, 2-amino acetophenone, 4-vinyl phenol, 4-vinyl-guaiacol, octanal, decanal and hexanal (Jezussek et al., 2002). However, 2AP is noted as to primary contributor to the grain aroma (Mo et al., 2019). Soil quality degradation poses a significant challenge to sustainable crop production, with heavy metal toxicity being a major factor in the decline of soil fertility (Keesstra et al., 2016; Iqbal et al., 2024). Heavy metals, particularly Cd, are among the most hazardous metals owing to their high toxicity and significant bioaccumulation in cereal plants (Singh et al., 2020). Whereas, rice, a food source for approximately 3.5 billion people globally (Dabral et al., 2019), is particularly vulnerable to Cd stress. Cd accumulation in arable soil is caused by industrial processes such as waste discharge, fertilization, mining, and smelting (Islam et al., 2017; Tang et al., 2019; Seleiman et al., 2020). Cd is more soluble and mobile than other metals, thus it is easily absorbed by plants, translocated, and deposited in various parts of plants (Chen et al., 2018; Adil et al., 2020). Furthermore, Cd is often not recyclable and difficult to eliminate from the soil, and it can transfer to cereal grains via the soil-plant-food cycle, posing a health risk to humans (Rizwan et al., 2016; Seleiman et al., 2020). In China, about 2.78 × 10^9^ m^2^ of farming land is contaminated by Cd (Xue et al., 2017; Huang et al., 2019). Cd inputs into the soil also adversely affect soil biodiversity and its associated ecosystem function (Haider et al., 2021). Thus, Cd has gained a lot of attention in arable soil because of its toxicity, accessibility, and long life in living organisms (Rizwan et al., 2016).

High levels of Cd in agricultural soils can detrimentally impact soil health, physio-chemical properties, and plant metabolism, leading to diminished crop growth, productivity, and quality (Mitra et al., 2018; Iqbal et al., 2023). Cd also hampers photosynthesis in plants and reduces the uptake of essential nutrients, thereby decreasing agricultural yields (Tran and Popova, 2013; Chen et al., 2018). Furthermore, Cd stress induces morpho-physiological and biochemical, alterations in plants, such as reduced root growth, stomatal density, and N metabolism enzyme activities (Bari et al., 2019; Huybrechts et al., 2020). The photosynthetic apparatus is particularly susceptible to Cd-induced damage, as chlorophyll production, crucial for photosynthesis, is compromised by Cd toxicity, impairing the photosynthetic process (Parmar et al., 2013; Li et al., 2010). Cd stress disrupts mitochondrial function in plants by disrupting redox balance and promoting the production of reactive oxygen species (ROS), which damage membrane lipids and alter metabolic activities (Chen et al., 2018; Huybrechts et al., 2020). The ROS produced under stress conditions are responsible for cellular oxidative damage and genotoxicity (Khan et al., 2022). Consequently, Cd, one of the most hazardous contaminants, requires particular attention to control its mobility in agricultural soils. Rice, a staple cereal crop for approximately 3.5 billion people globally (Dabral et al., 2019), is particularly vulnerable. A significant portion of Cd in the food chain originates from agricultural products, as Cd accumulates in soil plants via roots and enters the food supply, posing health risks to human immune, neurological, and reproductive systems (Parmar et al., 2013; Adil et al., 2020).

The utilization of organic amendments such as cattle manure, biochar, and compost represent an eco-friendly strategy for preventing Cd contamination (Gu et al., 2019; Hamid et al., 2020). However, these methods are often deemed impractical due to their associated costs and the potential introduction of additional pollutants (Pramanik et al., 2018). Vermicompost (VC), a nutrient-dense fertilizer, has emerged as a prevalent choice for rehabilitating metal-polluted agricultural soils (Wang et al., 2018; Zhang et al., 2020). VC is not only environmentally friendly but also a non-toxic amendment that enriches soil with essential nutrients and growth-promoting substances. In a systematic review by Oyege and Balaji Bhaskar (2023), it was reported that the supplementation of solo VC into agricultural systems enhances soil quality including enhanced permeability, aeration, drainage, and water-holding capacity and improves microbial biodiversity, ultimately boosting crop yield. Additionally, VC has been shown converting unavailable nutrients into available forms and supplies micro and macronutrients to plants, as well as it has elevated sulfur level than mineral fertilizer that can further enhance plant growth (Hoque et al., 2022; Shen et al., 2022). Alam et al. (2020) demonstrated that VC is superior to spent mushroom and organic fertilizers in mitigating the accumulation and uptake of Cd and other metals in plants. The maximum Cd^2+^ absorption rate of 170.70 mg g^-1^ by VC suggests its potential as an *in-situ* sorbent for Cd-treated soils (Zhu et al., 2017). In addition, VC is more effective than plant compost in reducing heavy metal levels in soil and its uptake in plants due to its maximum capacity, high specific area, strong cation exchange capacity, and enrichment in the active structural group (Li et al., 2021). Furthermore, it application can influence soil physical and biochemical properties, altering the chemical speciation of Cd in the soil (Wang et al., 2018) and increased the soil pH, thereby decreasing Cd bioavailability (Rafiq et al., 2014). Cd availability is sensitive to soil pH and exhibits a negative correlation with it. In alkaline conditions, Cd is present as CdHCO_3_^+^ or CdCO_3_ forms that are less bioavailable (Sauvé et al., 2000; Khaokaew et al., 2011; Shahid et al., 2017). Post-application, VC contributes polysaccharides, and mucilage from earthworms and microbes, and enhances soil physical structure, including aeration, porosity, aggregate stability, and drainage, all of which promote crop root development and nutrient uptake (Lim et al., 2015). VC is also a substantial source of both micronutrients and macronutrients for plants, leading to improved soil mineral content and increased plant growth and yield (Maji et al., 2017; Dubey et al., 2020). However, there is a lack of research assessing the impact of VC on paddy soil characteristics, the uptake of Cd by fragrant rice, the physiological and oxidative stress defense mechanisms, and rice yield under Cd stress.

This study explored the potential of VC as a soil conditioner for remediating Cd-polluted soils. The research focused on aromatic rice cultivars MXZ-2 and XGZ, which are popular in southern China for their desirable taste and flavor (Luo et al., 2020; Zhang et al., 2022). As a semi-aquatic tropical species grown in flooded fields, rice is particularly susceptible to Cd uptake and accumulation in its tissues (Wu et al., 2014). The main objectives of this study were (1) to investigate the effect of VC on the chemical properties of paddy soil, plant physiological attributes, and grain yield under Cd stress, (2) to determine the influence of VC on Cd accumulation and biochemical parameters in aromatic rice, including N metabolism related enzyme activities, N assimilation, and grain 2-acetyl-1-pyrroline (2AP) content, and (3) to elucidate the relationship between soil fertility, plant physiological processes, N assimilation, grain yield, and 2AP production in aromatic rice. We hypothesized that VC application could enhance soil health, reduces Cd accumulation in aromatic rice, thereby improve growth, quality, and yield. The findings are anticipated to contribute to establish a conceptual framework for safe and sustainable crop production in Cd-contaminated agricultural lands.

## 2 Materials and methods

### 2.1 Experimental location and soil properties

A pot experiment was conducted at South China Agricultural University in Guangzhou, China. The paddy soil from the specified rice field (0-15 cm depth) exhibited mild acidity, with a pH of 5.88. Additionally, the soil contained 0.93 g kg^-1^ of total phosphorus (TP), 140.45 mg kg^-1^ of available N (AN), and 1.19 g kg^-1^ of total N. Further details regarding the soil characteristics are provided in **Supplementary Table S1**.

### 2.2 Experimental details

In the current study, two aromatic rice varieties i.e., MXZ-2 and XGZ, showed differential responses to Cd stress were used. These cultivars were collected from the College of Agriculture, South China Agricultural University. The experiment was conducted using a complete block design having three replications during the late growing season (July-November) of 2023. Each plastic pot was filled by 15 kg of paddy soil collected from an unpolluted rice field at a depth of 15 cm. Four levels of VC were tested: VC1 = 0, VC2 = 2 t ha^-1^, VC3 = 4 t ha^-1^, and VC4 = 6 t ha^-1^, in conjunction with two Cd doses (Cd1 = 0 and Cd2 = 25 mg Cd kg^-1^ soil). The experiment comprised eight treatments: (1) Cd1VC1 = 0 Cd + 0 VC, (2) Cd1VC2 = 0 Cd + 2 t ha^-1^ VC, (3) Cd1VC3 = 0 Cd + 4 t ha^-1^ VC, (4) Cd1VC4 = 0 Cd + 6 t ha^-1^ VC, (5) Cd2VC1 = 25 mg Cd kg^-1^ soil + 0 VC, (6) Cd2VC2 = 25 mg Cd kg^-1^ soil + 2 t ha^-1^ VC, (7) Cd2VC3 = 25 mg Cd kg^-1^ soil + 4 t ha^-1^ VC, and (8) Cd2VC4 = 25 mg Cd kg^-1^ soil + 6 t ha^-1^ VC. Cd and VC were thoroughly mixed before seedling transplantation. The Cd treatment concentration was chosen based on previous studies in our lab (Kanu et al., 2017; Imran et al., 2021). The Cd used were CdCl_2_.2.5H_2_O, which was purchased from Sigma Aldrich, China. The seeds of two fragrant rice cultivars were cultivated in a plastic-trays, with each tray containing three hills. After 24 d, the uniform-sized seedlings were then transplanted into pots in mid-July, and the rice crops were harvested in November. The NPK fertilizer application rate was 300:150:300 (kg ha^-1^), with 1.80 g of N as urea, 0.90 g of P_2_O_5_ as superphosphate, and 2.20 g of KCl used. Uniform flooding irrigation was maintained from the time of seedling planting until physiological maturity.

### 2.3 Soil and plant samplings and analyses

#### 2.3.1 Soil chemical characteristics

A core sampler was utilized to extract soil samples from a depth of 15 cm, before the planting of seedlings and post-harvest. The collected soil samples were subsequently divided into two distinct parts: one portion was allocated for soil nutrient assessment, while the other was designated for molecular analysis (data are not provided in this study) and preserved at -80°C. Soil organic carbon (SOC) was analyzed using the oxidation method with K_2_Cr_2_O_7_-H_2_SO_4_, as described by Wang et al. (2003). For TN analysis, 200 mg soil samples were weighted using salicylic acid-sulfuric acid hydrogen peroxide according to Ohyama (1991), and finally, TN was calculated according to the micro-Kjeldahl technique recommended by Jackson (1956). In addition, soil pH, AN, total P, and total K were measured using the techniques detailed by Lu (2000).

#### 2.3.2 Gas exchange attributes and N metabolism related enzyme activities

On the seventh day of the heading stage, gas exchange attributes, such as transpiration rate (*Tr*) and net photosynthesis (*Pn*) were measured at sunny conditions using a portable photosynthesis system (LI-6800 System; Li-COR) to assess leaf physiological activity. In addition, the activity of NiR and NR enzymes in rice spikelets tissue was quantified employing the NIR-2-G and NR-2-Y assay kits, respectively, supplied by Biotechnology Co. Ltd., China. Additionally, GS and GOGAT enzyme activities in spikelets tissue were determined using the GS-2-Y and GOGAT-2-Y assay kits from the same manufacturer.

#### 2.3.3 Detection of NO_3_^-^, NH_4_^+^, and total N in rice leaves

The leaves samples were homogenized in distilled water, cooked for 15 minutes, and filtered to determine NO_3_ **^-^** and NH_4_^+^ levels. The NO_3_ **^-^** concentrations in leaves were determined using a previous approach (Cataldo et al., 1975). Whereas, the NH_4_^+^ content was measured using the Nessler reagent method (Molins-Legua et al., 2006). In addition, total N concentration was determined by using Kjeldahl method, as described by Barbano et al. (1990).

#### 2.3.4 Assessment of proline, total protein and amino acid contents

The soluble protein was measured in leaves by bovine serum albumin according to Bradford technique (Bradford, 1976). In addition, the proline content in leaves tissues was calculated according to the procedure of Bates et al. (1973). Total amino acid content in rice leaves was measured as described in the previous procedure (Barbano et al., 1990).

#### 2.3.5 Cd content determination in rice shoots and grains

The shoot and grain samples were dried, ground, and subsequently processed using a 4:1 (v/v) mixture of HNO_3_ **^-^** and HClO_4_. Following dilution to a final volume of 25 mL, Cd levels in these tissues were analyzed using a flame atomic absorption spectrometer, by the methodology previously described by Cao et al. (2014).

#### 2.3.6 Determination of grain 2AP contents

The 2AP levels in grains of two fragrant rice cultivars were determined by a synchronized distillation and extraction procedure and a Gas Chromatograph Mass Spectrometer according to the procedure of Mo et al. (2019).

#### 2.3.7 Grain yield and yield traits

Two fragrant rice cultivars were tested for grain yield and yield features. The rice grains were sun-dried to a moisture level of 12-14%. During the reproductive stage, the number of productive tillers was recorded. To determine the thousand-grain weight, 1000 rice grains were taken and weighed.

#### 2.3.8 Statistical analysis

All the data were collected in Microsoft Office Excel 2023 and all the data were showed as mean + standard errors. The collected data were subjected to one-way analysis of variance (ANOVA) for completely randomized designs, using Statistix 8.1 software (Analytical Software, Tallahassee, FL, USA). Before statistical analysis, the results were normalized using the arcsine transformation. Significant differences among the groups means for variables significantly affected by experimental factors were determined by Tukey’s HSD test at P < 0.05. Figures were prepared with OriginPro 2021.

## 3 Results

### 3.1 Influence of VC amendments on soil quality related parameters under Cd stress

The application of VC significantly (*p* < 0.05) improved various soil attributes such as TN, AN, SOC, pH, TK and TP, in soil treated with Cd- (, as detailed in **Table 1**. The VC supplementation alleviated the adverse effects of Cd on paddy soil health, with the most pronounced effect observed in all evaluated parameters at high VC amendments. Among the treatments, pots without Cd treatment (Cd1 + VC3) had higher values of soil quality attributes (i.e., pH, TN, AN, and SOC), whereas the soloCd2 pots had the lowest values. In comparison to the Cd2 alone treatment, the VC amendment treatments (Cd2 + VC3) enhanced the soil SOC, AN, TN and pH by 37.45%, 42.98%, 23.95% and pH 23.95% respectively. Likewise, lower VC inputs also increased each examined parameters, albeit to a lesser extent than the corresponding VC amendments under Cd stress.

**Table 1:** Influence of VC additions on soil fertility under Cd stress condition.

Treatment	pH	SOC (g kg^-1^)	TN (g kg^-1^)	AN (mg kg^-1^)	TP (g kg^-1^)	TK (g kg^-1^)
Cd1VC1	5.92 ± 0.86 e	12.24 ± 1.46 e	1.12 ± 0.09 d	143.50 ± 12.52 d	0.94 ± 0.04 d	16.40 ± 1.04 c
Cd1VC2	6.15 ± 0.52 b	15.32 ± 1.22 c	1.27 ± 0.08 b	155.08 ± 15.65 b	1.14 ± 0.02 b	18.20 ± 2.02 b
Cd1VC3	6.26 ± 0.60 a	17.42 ± 2.12 a	1.29 ± 0.08 a	178.50 ± 14.76 a	1.19 ± 0.04 a	23.30 ± 2.15 a
Cd1VC4	6.28 ± 0.66 a	17.46 ± 2.12 a	1.30 ± 0.10 a	184.40 ± 12.14 a	1.18 ± 0.06 a	24.45 ± 2.15 a
Cd2VC1	5.60 ± 0.74 d	11.22 ± 1.66 d	0.96 ± 0.05 e	109.12 ± 10.70 e	0.92 ± 0.03 d	13.30 ± 1.05 d
Cd2VC2	6.01 ± 0.44 c	13.24 ± 1.70 d	1.09 ± 0.07 c	152.52 ± 21.50 c	0.99 ± 0.02 c	14.40 ± 1.12 d
Cd2VC3	6.14 ± 0.54 b	15.42 ± 2.20 b	1.19 ± 0.10 b	156.04 ± 17.54 b	1.12 ± 0.04 b	18.50 ± 1.42 b
Cd2VC4	6.15 ± 0.50 b	15.99 ± 2.22 b	1.20 ± 0.09 b	161.66 ± 15.50 b	1.13 ± 0.03 b	19.44 ± 1.22 b

Cd, Cadmium; VC, vermicompost; SOC, soil organic carbon; TN, total nitrogen; AN, available nitrogen; TP, total phosphorous; TK, total potassium; Cd1VC1 = 0 mg Cd + 0 t ha^-1^ VC; Cd1VC2 = 0 mg Cd + 2 t ha^-1^ VC; Cd1VC3 = 0 mg Cd + 4 t ha^-1^ VC; Cd1VC4 = 0 mg Cd + 6 t ha^-1^ VC; Cd2VC1 = 25 mg Cd + 0 t ha^-1^ VC; Cd2VC2 = 25 mg Cd + 2 t ha^-1^ VC; Cd2VC3 = 25 mg Cd + 4 t ha^-1^ VC; Cd2VC4 = 25 mg Cd + 6 t ha^-1^ VC. Tukey checks were used to assess the means. Statistics reveal that the values in the column with similar letters are statistically (*p* < 0.05) the same. The lettering was assigned after applying Tukey HSD test at 5%.

### 3.2 Influence of VC on plant net photosynthesis and transpiration rate under Cd treatment

**Figure 1:**
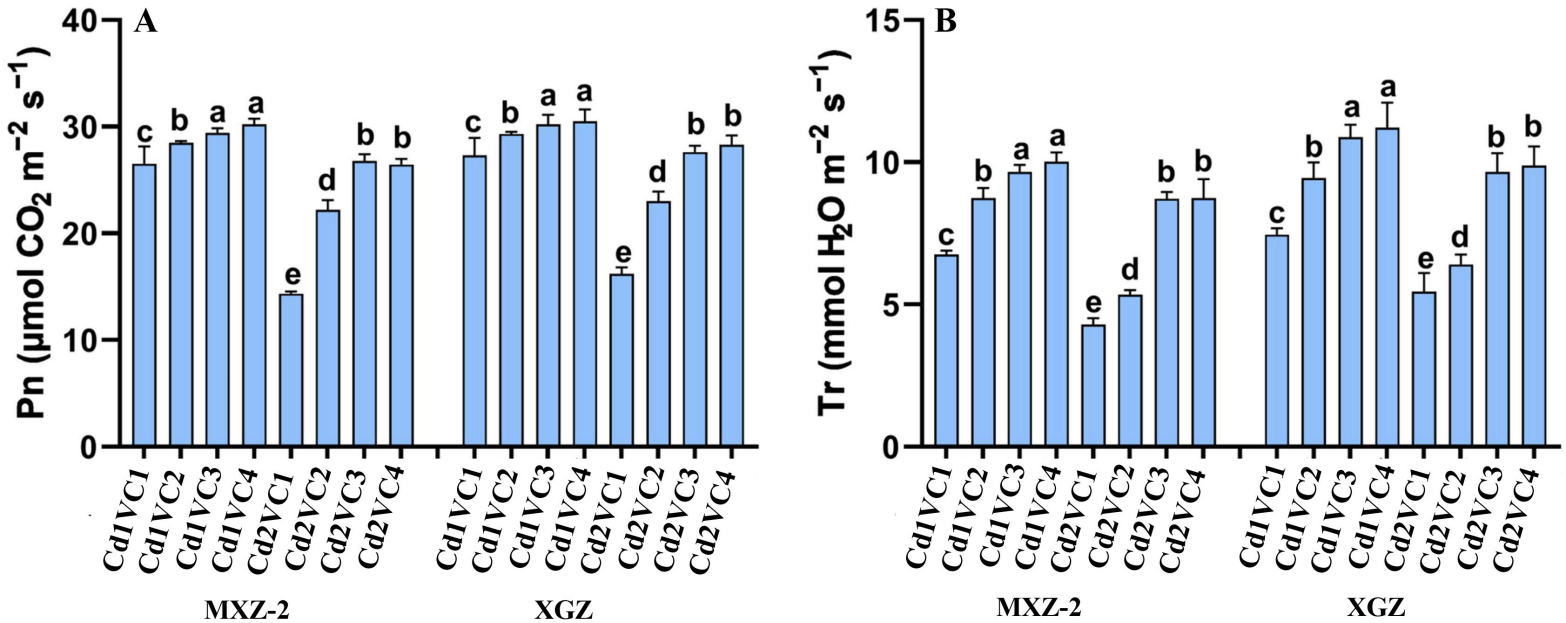
Effect of VC application on *Pn* **(A)** and *Tr* rate **(B)** of two fragrant rice cultivars (MXZ-2 and XGZ) at heading stage subjected to Cd treatment. Tukey HSD test were applied to relate the means of the treatments. Bars with distinct letter combinations show significant differences at (*P* < 0.05). See **Table 1** for treatment details.

The study examined the impact of VC application on the photosynthetic parameters of two fragrant rice varieties, such as XGZ and MXZ-2, under Cd stress condition. Significant variations in photosynthesis were observed, as shown in **Figure 1**. In plants subjected to Cd stress, VC supplementation improved photosynthetic parameters, including net *Pn* and *Tr*, during the reproductive phase. Both cultivars exhibited a comparable response to the treatments. Specifically, the Cd2 + VC3 treatment led to an 86.75% increase in *Pn* and a 102.20% increase in *Tr* for MXZ-2, and a 70.05% and 81.22% increase for XGZ, respectively, compared to the Cd2 + VC1 treatment, as illustrated in **Figure 1**. Additionally, low doses of VC significantly enhanced physiological activity in the leaves of fragrant rice under Cd treatment conditions.

### 3.3 Influence of VC application on N metabolism related enzymes

**Figure 2:**
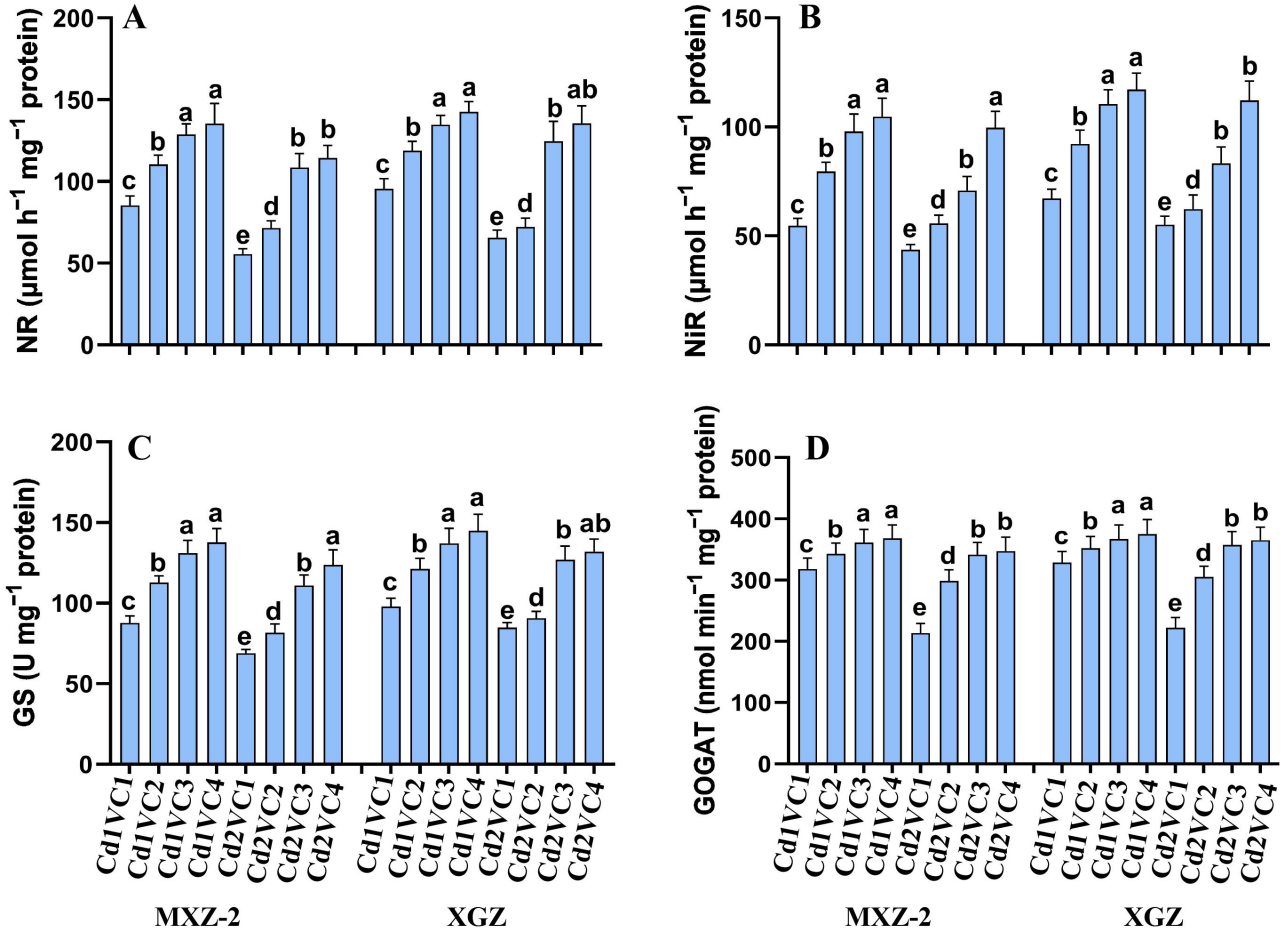
Effect of VC on N metabolism enzyme activities, **(A)** nitrate reductase, **(B)** nitrite reductase, **(C)** glutamine synthetase, and **(D)** glutamate oxoglutarate aminotransferase in spiklets of two fragrant rice cultivars (MXZ-2 and XGZ) subjected to Cd treatment. Tukey HSD test were applied to relate the means of the treatments. Error bars are standard errors of the mean. At P < 0.05, bars with distinct letter combinations show significant differences. See **Table 1** for treatment details.

The study examined the impact of VC supplementation on N assimilation and accumulation in plants subjected to Cd toxicity (**Figure 2**). Enzyme activities associated with N metabolism, including NiR, NR, GOGAT, and GS, were notably diminished under Cd treatment compared to non-Cd exposed plants. Cultivar variations were observed, with XGZ showing a less pronounced decline in enzyme activities, suggesting greater tolerance to Cd stress. Interestingly, VC supplementation mitigated Cd treatment, particularly under high VC treatments, and enhanced the activities of N metabolism enzymes in leaves. Both cultivars exhibited a similar response pattern to the treatments. Specifically, the Cd2 + VC3 treatment led to a substantial increase in NR (95.66% and 90.05%), NiR (62.15% and 50.50%), GS (61.10% and 49.66%), and GOGAT (58.45% and 60.78%) activities in MXZ-2 and XGZ, respectively, compared to plants treated with Cd alone. Furthermore, plants treated with lower concentrations of VC displayed significantly higher N metabolism enzyme activities than those exposed solely to Cd treatment.

### 3.4 Effect of VC addition on plant inorganic and total N content

**Figure 3:**
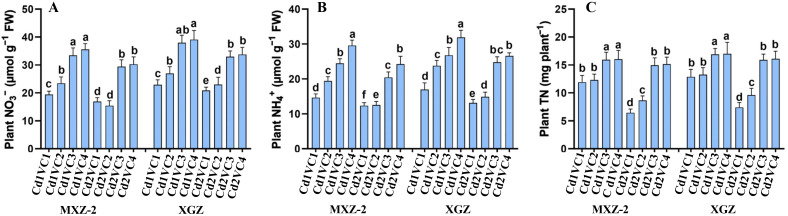
Effect of VC application on plant inorganic N and total N content such as NO_3_^-^ **(A)**, NH_4_^+^ **(B)** and TN **(C)** in leaves of two fragrant rice cultivars (MXZ-2 and XGZ) subjected to Cd treatment. Tukey HSD test were applied to relate the means of the treatments. Error bars are standard errors of the mean. At *P* < 0.05, bars with distinct letter combinations show significant differences. See **Table 1** for treatment details.

The study revealed that Cd stress significantly reduced the levels of NO_3_ **^-^**, NH_4_^+^, and TN content in aromatic rice cultivars, as depicted in **Figure 3**. Conversely, the supplementation of VC mitigated the detrimental impact of Cd stress on rice plants, enhancing both inorganic N and total N content. Notably, both cultivars exhibited a comparable response to the treatments. The lowest concentrations of NO_3_^-^, NH_4_^+^, and TN were observed in plants subjected to the Cd2 + VC1 treatment. Among the treatments, the Cd2 + VC3 treatment led to a substantial increase in NO_3_^-^ (74.44% and 58.05%), NH_4_^+^ (65.50% and 88.66%), and plant TN content (147.50% and 123.85%) in MXZ-2 and XGZ, respectively, compared to plants treated with Cd2 + VC1. Furthermore, plants in pots treated with lower concentrations of VC demonstrated significantly higher N metabolism enzyme activity than those subjected to Cd treatment alone.

### 3.5 Role of VC addition in proline, protein, and amino acid production

The production of soluble proteins, proline, and amino acids in leave were varied significantly between cultivars under Cd treatment when treated with VC, as indicated in **Table 2**. In Cd-treated plants, proline levels were markedly elevated compared to those in non-Cd-treated plants. However, VC application mitigated Cd-induced stress and decreased proline synthesis. Specifically, proline content was decreased by 30.74% and 23.25% in the XGZ and MXZ-2 cultivars, respectively, in the Cd2 + VC3 treatment compared to the Cd2 + VC1 treatment. Similarly, a lower concentration of VC significantly reduced proline content in Cd-treated plants. Moreover, the supplementation of VC alleviated Cd stress in fragrance rice, significantly (*p* < 0.05). For instance, compared to the Cd2 + VC1 treatment, the Cd2 + VC3 treatment increased leaf total protein and amino acid content by 25.95% and 29.62% in XGZ, and by 68.70% and 49.50% in MXZ-2, respectively. Additionally, the results indicated that MXZ-2 exhibited lower proline and soluble protein content than XGZ, suggesting that MXZ-2 may be more susceptible to stress conditions.

**Table 2:** Influence of VC on fragrant rice biochemical attributes and Cd accumulations under Cd stress condition.

Genotype	Treatment	Proline content (µg g^-1^ FW)	Soluble protein (mg g^-1^ FW)	Amino acid (µg g^-1^ FW)	Cd content (µg g^-1^ DW)		2AP (ng g^-1^ FW)
					Shoot	Grain	Grain
	Cd1VC1	18.35 ± 1.82 d	404.18 ± 12.33 b	24.24 ± 1.52 c	10.85 ± 0.67d	0.14 ± 0.01 d	299.18 ± 12.33b
	Cd1VC2	18.86 ± 2.01 d	406.75 ± 18.24 b	26.3 ± 2.02 b	9.88 ± 0.78 e	0.12 ± 0.02 e	301.75 ± 14.24 b
**MXZ-2**	Cd1VC3	13.85 ± 1.45 e	423.32 ± 18.45 a	28.22 ± 1.72 a	8.42 ± 0.65 f	0.09 ± 0.01f	321.32 ± 18.45 a
	Cd1VC4	14.04 ± 1.05 e	434.04 ± 15.05 a	28.44 ± 2.12 a	8.76 ± 0.60 f	0.08 ± 0.01f	329.04 ± 15.05 a
	Cd2VC1	21.23 ± 1.67 a	316.45 ± 14.45 d	15.34 ± 0.82 e	42.85 ± 3.45 a	1.56 ± 0.05 a	168.05 ± 14.45 d
	Cd2VC2	18.34 ± 1.44 b	375.44 ± 16.66 c	17.34 ± 1.12 d	36.96 ± 1.10 b	0.98 ± 0.05 b	270.40 ± 12.66 c
	Cd2VC3	16.24 ± 2.22 c	398.28 ± 12.68 b	25.88 ± 2.80 b	20.86 ± 2.88 c	0.66 ± 0.02 c	296.28 ± 12.68 b
	Cd2VC4	16.54 ± 1.25 c	408.27 ± 15.08 b	26.44 ± 1.52 b	18.88 ± 2.85 c	0.67 ± 0.02 c	303.25 ± 10.08 b
	Average	17.18 b	400.84 b	26.44 a	19.75 a	0.51 a	284.72 b
	Cd1VC1	21.65 ± 1.20 c	410.34 ± 14.45 d	25.44 ± 1.50 c	8.88 ± 0.86 d	0.15 ± 0.02 d	317.50 ± 10.35 b
	Cd1VC2	18.16 ± 1.22 d	428.94 ± 18.34 c	27.28 ± 2.60 b	7.12 ± 0.88 e	0.21 ± 0.02 e	320.08 ± 11.25 b
	Cd1VC3	17.90 ± 1.55 d	467.55 ± 14.22 b	29.20 ± 2.85 a	6.45 ± 0.44 f	0.04 ± 0.01f	336.65 ± 12.30 a
**XGZ**	Cd1VC4	18.32 ± 1.75 d	487.54 ± 16.25 a	30.22 ± 2.20 a	6.65 ± 0.45 f	0.10 ± 0.01f	340.37 ± 15.30 a
	Cd2VC1	24.52 ± 2.23 a	324.68 ± 17.78 e	18.33 ± 1.05 e	40.84 ± 3.40 a	1.25 ± 0.04 a	172.68 ± 10.44 d
	Cd2VC2	21.63 ± 1.96 b	399.23 ± 12.20 d	22.22 ± 1.80 d	30.98 ± 2.18b	0.96 ± 0.03 b	288.77 ± 8.33 c
	Cd2VC3	19.95 ± 1.45 c	420.46 ± 15.44 c	27.98 ± 1.80 b	20.88 ± 1.80 c	0.71 ± 0.02 c	311.68 ± 12.33 b
	Cd2VC4	20.22 ± 168 c	422.46 ± 15.44 c	28.04 ± 2.80 b	19.98 ± 1.80 c	0.71 ± 0.02 c	321.62 ± 14.33 b
	Average	20.3 a	420.9 a	26.62 a	16.72 b	0.47 b	302.05 a
	Treatment (T)	**	**	**	**	**	**
	Cultivar (C)	*	*	ns	*	*	*
	T × C	ns	ns	ns	ns	ns	ns

Cd, cadmium; 2AP, 2-acetyl-1-pyrroline; XGZ, Xiangyaxiangzhan; MXZ-2, Meixiangzhan-2; ns=non-significant; **& * are significant at 1% & 5%, respectively. Results are the averages of three replications, and Tukey tests were used to compare the treatment mean. The lettering was assigned after applying Tukey HSD test at 5%. The proline content, total protein, and amino acid content were determined in leaves of two rice cultivars. See **Table 1** for treatment combination details.

### 3.6 Cd accumulation in fragrant rice under VC application

The uptake and accumulation of Cd in the shoots and grains of two fragrant rice varieties were significantly elevated under Cd-treated soil conditions (**Table 2**). However, the application of VC declined Cd-related toxicity and substantially diminishes Cd uptake in rice tissue. The Cd content in rice tissue exhibits the following trend: shoot > grains.

High level of VC amendments, particularly Cd1 + VC4, resulted in the lowest Cd accumulation in rice tissue. The application of VC significantly decreased Cd concentrations in both leaves and stems compared to the Cd2 treatment alone. Specifically, Cd2 combined with VC3 decreased Cd accumulation by 105.50% in the shoots and 136.66% in the grains of the MXZ-2 rice cultivar, while in the XGZ cultivar, reduction were 95.55% in shoots and 100.20% in grains. These data indicate that a high dosage of VC significantly diminishes Cd accumulation in rice plants. Furthermore, the results suggest that the XGZ cultivar exhibits greater resistance to Cd than the MXZ-2 cultivar.

### 3.7 Effect of VC additions on 2AP content

The Cd-stressed plants significantly declined the 2AP content in rice grains. However, VC supply mitigated the Cd-induced toxicity and significantly improved the grain 2AP content (**Table 2**). Of the treatments, higher 2AP content was observed in the Cd1 + VC4 treated plants. All the treatments followed a similar pattern for both cultivars. Whereas, the lowest 2AP content in grains was noted in the Cd2 treatment only. Relative to alone Cd2 treatment, high VC supplementation (VC3) treatment into Cd-stressed plants enhanced the 2AP content by 76.50% and 80.46% in XGZ and MXZ-2 cultivars, respectively. Similarly, low VC treatment also increased the 2AP content in rice grains under Cd stress condition.

### 3.8 Effect of VC supply on yield and grain yield attributes

The Cd treatment significantly decreased the yield and yield traits of both varieties (**Table 3**). Nevertheless, VC additions counteracted the Cd stress and significantly improved grain yield and yield attributes. Interestingly, the treatments followed a similar pattern for both cultivars, and higher grain yield was noted in the Cd1 + VC4 treatment. Related to Cd-only plants, VC input treated plants, i.e., Cd2 + VC3 enhanced the productive tillers, thousand-grain weight, and yield by (53.22% and 49.50%) and (60.05%, and 77.55%) in XGZ and MXZ-2 cultivars, respectively. Similarly, other low VC doses also increased grain yield and yield attributes in a Cd-treated condition.

**Table 3:** Influence of VC supply on rice cultivars on yield and yield traits in a Cd-stressed soil.

Genotype	Treatments	No. of tillers (pot^-1^)	Productive tillers (pot^-1^)	Grains panicle^-1^	1000 GW (g)	Grain yield (g pot^-1^)
	Cd1VC1	28 ± 1.22 d	20 ± 1.40 e	148 ± 10.23 c	16.45 ± 0.87 c	83.08 ± 4.65 d
	Cd1VC2	36 ± 1.89 b	30 ± 2.42 b	165 ± 13.50 b	18.54 ± 1.26 b	102.85 ± 8.85 c
	Cd1VC3	44 ± 3.24 a	35 ± 2.04 a	186 ± 16.08 a	20.05 ± 1.34 a	116.34 ± 8.45 b
**MXZ-2**	Cd1VC4	45 ± 3.54 a	35 ± 2.25 a	188 ± 15.08 a	21.25 ± 1.55 a	122.35 ± 9.40 a
	Cd2VC1	22 ± 1.20 e	18 ± 2.23 f	126 ± 12.32 e	12.35 ± 1.05 d	61.45 ± 6.77 e
	Cd2VC2	20± 2.11 d	24 ± 1.88 d	161 ± 9.76 d	16.55 ± 1.02 c	73.44 ± 9.65 d
	Cd2VC3	35 ± 2.66 bc	28 ± 2.44 b	172 ± 16.22 b	18.96 ± 2.22 b	98.34 ± 8.24 c
	Cd2VC4	36 ± 2.62 b	28 ± 2.44 b	175 ± 14.25 b	19.20 ± 2.52 b	100.32 ± 7.55 c
	Average	33.25 b	27.3 b	156.10 b	17.10 b	94.75 b
	Cd1VC1	32 ± 1.42 d	22 ± 1.20 e	154 ± 8.23 c	17.40 ± 1.02 c	92.08 ± 3.62 d
	Cd1VC2	39 ± 1.86 b	33 ± 2.42 c	171 ± 12.50 b	20.50 ± 1.22 b	115.88 ± 6.80 c
**XGZ**	Cd1VC3	49 ± 2.25 a	36 ± 2.04 b	192 ± 14.08 a	22.06 ± 1.35 a	122.30 ± 8.48 b
	Cd1VC4	51 ± 3.14 a	38 ± 2.25 a	191 ± 12.08 a	23.22 ± 1.05 a	129.30 ± 9.15 a
	Cd2VC1	23 ± 1.20 e	20 ± 1.44 f	129 ± 10.30 e	13.34 ± 0.85 d	64.40 ± 6.77 f
	Cd2VC2	25 ± 2.15 d	25 ± 1.88 d	169 ± 9.78 d	17.54 ± 1.52 c	76.44 ± 7.62 e
	Cd2VC3	39 ± 2.06 bc	33 ± 2.42 c	174 ± 10.22 b	19.96 ± 1.82 b	114.34 ± 7.66 c
	Cd2VC4	39 ± 2.62 b	34 ± 2.44 c	179 ± 14.25 b	20.14 ± 2.02 b	119.32 ± 8.25 c
	Average	37.12 a	31.25 a	169.90 a	19.30 a	104.25 a
	ANOVA					
	Treatment (T)	**	**	**	**	**
	Cultivar (C)	*	*	*	*	*
	T × C	ns	ns	ns	ns	ns

Cd, cadmium; GW, grain weight; XGZ, Xiangyaxiangzhan; MXZ-2, Meixiangzhan-2; ns=non-significant; **& * are significant at 1% & 5%, respectively. Results are the averages of three replications, and Tukey checks were used to compare the treatment mean. The lettering was assigned after applying Tukey HSD test at 5%. See **Table 1** for treatment combination details.

## 4 Discussion

Heavy metals, specifically Cd, often disrupt soil fertility, reduces soil pH and affect variety of morphological, and physio-biochemical characteristics of plants including growth, photosynthesis, N assimilation, and accumulation (Hussain et al., 2020). Therefore, mitigating Cd stress in soil and its impact on plant growth, and development is a crucial objective for plant researchers. One effective and environmentally friendly approach is *in situ* stabilization, which involves immobilizing Cd through the application of organic fertilizers such as cattle dung, VC, and biochar (Hamid et al., 2020; Iqbal et al., 2024). In this study, we investigated the effect of VC application on the chemical properties of paddy soil, as well as the physiological and biochemical characteristics of plants, 2AP content in grains, and the yield of fragrance rice grown under Cd stress condition.

### 4.1 VC amendment improved the Cd-contaminated soil properties

The current investigation, reveals that the application of VC significantly improves the quality of soil affected by Cd stress. Our findings suggest that increasing the biodegradation rate of VC enhances soil quality by gradually releasing essential nutrients for plant uptake. We observed a notable increase in soil pH with the addition of VC compared to untreated and Cd-treated soil. In contrast, Ni et al. (2018) noted that nitrification, resulting from the heavy use of synthetic N fertilizers, produces hydrogen ions (H^+^), which subsequently reduces soil pH. The acidic nature of synthetic N may contribute to this pH reduction, as posited by Adekiya et al. (2020). Our study indicates that Cd2 alone treatment also enhanced the soil acidity, compared to VC application treatments. These differences in Cd accumulation in rice grains in different soils may be attributed to disparities in Cd phytoavailability, which is influenced by soil pH. Specifically, lower soil pH levels correlate with increased Cd accumulation in rice grains, and conversely, higher pH levels result in reduced Cd uptake (Rafiq et al., 2014). Furthermore, Ok et al. (2011a; b) observed similar trends in rice cultivated in Cd-contaminated soils where amendments that altered soil pH were applied. In line with our findings, previous studies have also noted low pH levels in the presence of Cd toxicity (Jin et al., 2020). Conversely, the addition of organic N has been shown to substantially decrease soil acidity (Iqbal et al., 2019). Correspondingly, our research indicates that VC supplementation markedly increases soil pH. Hydroxyl ions (OH-) are generated from charged functional groups in organic additives and the hydrolysis of CaCO_3_ interacts with H^+^ ions, thereby increasing the soil pH. These hydroxyl ions encompass phenolic, hydroxyl, and carboxyl groups (Gul et al., 2015). The majority of plant species thrive in soil with a pH adjusted to near neutrality by the application of VC (Fernandez-Bayo et al., 2009).

**Figure 4:**
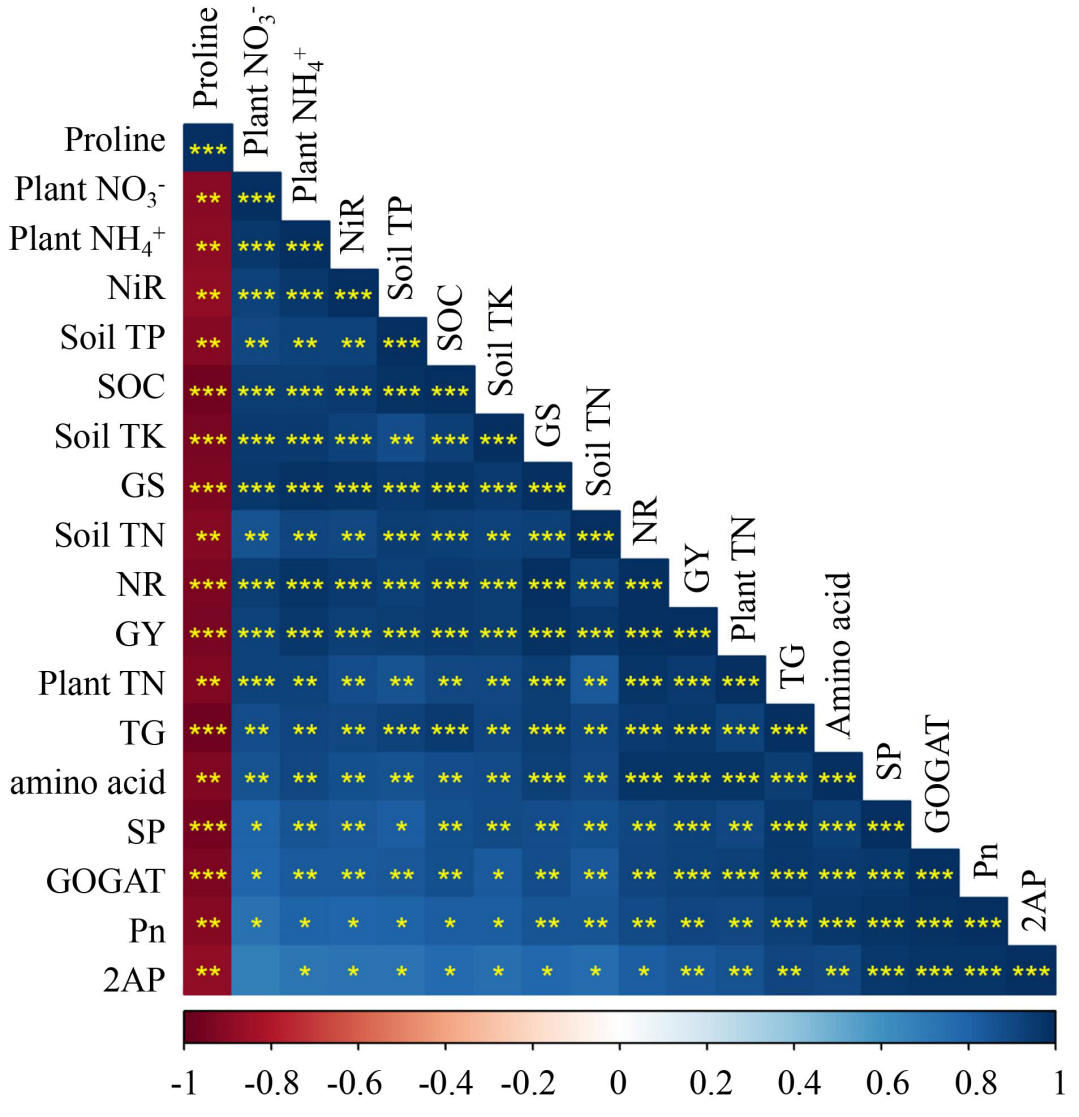
The correlation analysis between soil chemical traits, plant physiological, biochemical, and grain yield of rice under Cd toxicity to the application of different VC amendments. 2AP (2-acetyl-1- pyrroline), Pn (net photosynthesis rate), NR (nitrate reductase), NiR (nitrite reductase), GS (glutamine synthetase), GOGAT (glutamate oxoglutarate aminotransferase), SP (soluble protein), TN (total nitrogen), GY (grain yield), TK (total potassium), TP (total phosphorous). Asterisks (*), (**), and (***), indicate significant differences at P < 0.05, P < 0.01 and P < 0.001 respectively.

The improvements in soil chemical properties observed with high-VC treatments in this study can be attributed to the compost rich organic matter content and its variety of essential plant nutrients (Tejada et al., 2010). Liang et al. (2017) noted that metals generally do not easily dissolve or move through soil with high pH. Consequently, the elevated pH of the soil following VC fertilizer application may have significantly contributed to the retardation of Cd migration in the present experiment, and the enhanced mineral content of the soil promotes robust plant growth. Furthermore, the improved mineral content of the soil ensures vigorous plant growth. Lim et al. (2015) reported that the VC application promotes the secretion of mucus by earthworms, polysaccharides, and microorganisms, which enhance the soil’s physical structure and are crucial for plant root development and nutrient absorption. These physical improvements encompass increased porosity, improved drainage, and aeration, and enhanced aggregate stability. Additionally, the soil chemical properties were positively correlated with plant physiological and biochemical characteristics, as well as the yield of fragrance rice grains (**Figure 4**). This analysis indicated that the enhancements in soil quality are directly linked to plant growth and development. In summary, the application of VC can enhance soil fertility and crop production while also mitigating the mobility of Cd in paddy soils.

### 4.2 VC amendments enhanced plant physiological and biochemical attributes

Photosynthesis is the main element of plant physiological activity and productivity by enhancing crop growth and biomass accumulation (Khan et al., 2017). In this experiment, the VC enhanced the plant’s photosynthetic efficiency compared to the Cd stress environment (**Figures 1**, **2**). The enhancement in leaf photosynthetic activity induced under VC application could be primarily attributed to the boosted soil fertility in the current work (**Table 1**), faster release of soil nutrients from VC in the early growth stages and gradual and slow release of crop-related nutrients from VC throughout the crop period (Yang et al., 2015; Iqbal et al., 2021). Photosynthesis experienced a strong reaction to water and soil health (Makoto and Koike, 2007). An adequate supply of water and nutrients reduces the concentration of water-soluble nutrients and mitigates the root-derived stress signal abscisic acid (ABA), leading to the opening of leaf stomata and enhancing their water potential and physiological activity (Daszkowska-Golec and Szarejko, 2013). Additional evidence is provided by the present study linear regression analysis, which indicates a highly significant correlation between soil chemical properties and leaf photosynthetic activity (**Figure 4**).

NO_3_^-^ and NH_4_^+^ are the primary forms of N that plants use for growth activities, and N uptake and assimilation is a vital metabolic activity that not only controls crop growth and development but also plays a critical role in crop survival in a polluted soil (Imran et al., 2019). In N assimilation, NO_3_^-^ is converted into NO_2_^-^ and NH_4_^+^ by the sequential activity of N metabolizing enzymes, NR and NiR, whereas NH_4_^+^ is directly converted into amino acids and protein by the concerted actions of GS and GOGAT (Balotf et al., 2016). In the current study, solo supplementation of VC increased the NO_3_^-^, NH_4_^+^ and total N maybe because VC application significantly influence the essential nutrient particularly N and P in the soil (Ramazanoglu, 2024). The application of VC has been shown to significantly influence plant growth through its substantial impact on key soil properties. Specifically, VC has been found to enhance processes such as mineralization and nitrification, thereby increasing the availability of essential mineral nutrients to plants compared to other organic fertilizers (Karlsons et al., 2016). Furthermore, studies indicated that the application of VC results in elevated levels of soil organic matter, microbial activity and N concentration, the latter being a critical nutrient for plant growth (Akhzari and Pessarakli, 2017; Erdal and Ekinci, 2020; Iqbal et al., 2024). These findings collectively underscore the potential of VC as a potent soil amendment for improvement of plant physiological and enzymatic activities. In contrast, Cd stress considerably reduced NR and NiR enzyme activities in plants (**Figure 2**). A possible explanation for this is that the Cd stress significantly reduced the soil fertility and NO_3_^-^ content in rice leaf tissues (**Table 1**, **Figure 3**). However, the VC addition increased the N metabolism enzyme activities, including NR and NiR. Interestingly, in the current work, VC supply also increased the GOGAT and GS activity and simultaneously enhanced amino acid and SP contents in Cd-stressed plants, leading to Cd tolerance mechanism (**Table 2**). The main reason for the improvements in N metabolism activities was mainly due to enhanced plant physiological activity in Cd-contaminated soils in the presence of VC.

In this work, Cd stress considerably reduced the total N in both cultivars (**Figure 3**). The main reason might be due to significantly decreased NO_3_^-^ level and N assimilation, N enzymes activities in the current study under Cd stress condition. Similarly, earlier research also stated that Cd stress reduced N accumulation (Campbell, 2001; Hussain et al., 2020). However, the VC supply decreased the side effects of Cd stress on soil health and crop growth and development and significantly (*p* < 0.05) increased the soil nutritive status and plant physio-biochemical activity under Cd-stressed conditions. This improvement in soil fertility and plant growth and physiological activity ultimately increased plant N uptake and assimilation in rice, and these VC-related side effects are nearly to be accomplished by enough NO_3_ **^-^** accumulation, upgrading the activity of N assimilation enzymes and accumulating nitrogenous compounds (i.e., amino acid and proline) in this study. Similarly, Sun et al. (2012), also stated that several N metabolism enzymes play a key part in plant N accumulation. In this work, the greater activity of N assimilation enzymes in VC-added plants. The N metabolism enzymes, i.e., GOGAT and GS in the reproductive stage were positively correlated to N assimilation and grain yield and quality. Ceusters et al. (2019), reported that soil N availability has a strong relationship with plant N uptake and enhanced N metabolism enzyme activities. Furthermore, the Cd stress reduced the 2AP level and yield of cultivars, while VC alleviated the Cd stress and enhanced the 2AP level and yield of fragrant varieties (**Table 2**). A possible explanation is that Cd stress reduced N metabolic activity and thus reduced plant TN content in different organs. However, VC amendments counteracted the Cd-related inhibitory effect on N assimilation and utilization by enough N uptake and strengthened N assimilation enzyme activities. In addition, the plant physiological and biochemical attributes were highly positively correlated with 2AP production in grains of both fragrant rice cultivars (**Figure 4**). This analysis revealed that the improvement in rice physiological attributes are closely linked to grain quality. In conclusion, the application of VC can improve the soil fertility and plant physicochemical attributes, which in turn positively impacts crop quality. Our findings are also in agreement with the previous findings indicating a strong positive correlation between a plant physiological and biochemical traits and both 2AP and grain yield of rice (Yang et al., 2012; Mo et al., 2019).

The cytoplasm of plants contains proline, a compound that modulates osmotic pressure by adjusting cellular water potential (Muneer et al., 2011). Our findings indicate that under Cd stress, the proline concentration in rice leaves significantly increases (**Table 2**). Elevated proline levels in plants are commonly associated with heavy metal stress, particularly Cd, as stressed plants exhibit increased resilience (Bauddh and Singh, 2012). The degradation of plant proteins may correlate with heightened proline levels, which could serve as an indicator of plant tissue damage (Elmer and White, 2018). Our results are consistent with Elmer and White (2018), who reported that increased protease activity leads to protein deficiency in response to Cd stress. However, the application of VC significantly enhanced protein content and 2AP in plant grains, counteracting the adverse effects of Cd stress (**Table 2**). Our study suggests that the addition of organic amendments to soil enhances its fertility, thereby promoting plant physiological and biochemical processes through the improved uptake and accumulation of essential nutrients. Furthermore, VC application reduced leaf proline content and bolstered plant defense mechanisms by enhancing physiological activity, demonstrating a mitigating effect on maintaining osmotic balance in plants exposed to Cd pollution.

### 4.3 VC application reduced the Cd accumulation in shoot and grains of rice plant

The current study demonstrates that the application of VC significantly reduces Cd uptake in plant organs such as shoots and grains, as shown in **Table 2**. This effect is primarily attributed to VC’s ability to decrease Cd’s accessibility and mobility. VC acts as a soil conditioner by promoting metal precipitation and complexation, thereby providing plants with nutrients and organic matter while concurrently diminishing the mobility and bioavailability of metals in the soil (Deng et al., 2017). Furthermore, due to its elevated cation exchange capacity and surface area, VC is considered a promising agent for stabilizing heavy metals in soil (Wang et al., 2018; Ding et al., 2021). Additionally, Wan et al. (2020) observed that the supplementation of organic fertilizers led to a substantial reduction in Cd levels in rice grains, ranging from 7.8 to 79.3%. Similarly, Tang et al. (2015) found that the addition of organic amendments reduced heavy metal content in the roots and shoots of plants.

### 4.4 Rice yield and yield traits

Cd stress significantly reduced the yield and yield components in both cultivars examined. Conversely, the application of VC markedly enhanced rice yields and yield components, as shown in **Table 3**. Enhancements in crop production are closely associated with enhanced soil fertility (Iqbal et al., 2022, 2024). Organic fertilizers improve soil quality and fertility, thereby promoting plant growth, development, quality, and yield (Iqbal et al., 2022). In this study, nutritional values of the soil were increased upon application of VC into the soil (**Table 1**), which facilitated aromatic physiological activity, growth, and yield by providing essential nutrients during the growth phase. This was corroborated by the correlation analysis conducted, which indicated that soil quality parameters were highly positively correlated with the plant’s physiological and biochemical attributes, ultimately influencing yield and yield characteristics (**Figure 4**). Iqbal et al. (2022) demonstrated that variations in rice yield are closely associated with soil fertility. In summary, the use of VC may enhance soil fertility and plant physical-biochemical attributes, directly impacting and improving crop productivity.

## 5 Conclusions

Our study offer novel insights into how VC supplementation enhances 2AP content and grain yield in rice under Cd stress by improving N assimilation and utilization. We found that Cd stress significantly impeded soil fertility and plant physiological processes, including the activities of N metabolism related enzymes (NR, NiR, GS, and GOGAT), as well as 2AP production and grain yield in rice. However, the application of VC effectively counteracted the Cd-induced decline in soil fertility and plant physio-biochemical characteristics, resulting in improved 2AP levels and grain yield in both rice cultivars studied. The results indicated that VC application immobilized Cd in paddy soil, enhancing soil quality and reducing Cd accumulation in rice tissues (shoots and grains), while also decreasing the uptake of essential nutrients from the soil. Our findings suggest that VC supplementation enhances plant physiological efficiency and biochemical status by mitigating the deleterious effects of Cd on plant health, potentially through decreased Cd uptake and improved leaf photosynthetic efficiency, soluble protein levels, and regulation of nitrogenous compounds such as amino acids and proline. As a result, our study demonstrates that VC amendments can improve N uptake and assimilation, leading to increased 2AP levels and grain yield in rice under Cd stress conditions.

## Data Availability

The original contributions presented in the study are included in the article/**Supplementary Material**, further inquiries can be directed to the corresponding author/s.
