# Supplementary material for: Vermicompost application improves leaf physiological activity, 2-acetyl-1-pyrroline, and grain yield of fragrant rice through efficient nitrogen assimilation under Cd stress
Source: Front Plant Sci. 2024 Dec 2;15:1481372. doi: 10.3389/fpls.2024.1481372 (PMC11646779; doi:10.3389/fpls.2024.1481372)
Supplement: Supplementary file 1 [file Table1.docx]

Supplementary Material

**Table S1**. Vermicompost and soil physiochemical parameters during the pre-experiment stage

| **Soil Properties** | **Content** |
| --- | --- |
| Soil organic carbon (g kg^-1^) | 9.05 |
| Total potassium (g kg^-1^) | 4.22 |
| Available phosphorus (mg kg^-1^) | 15.3 |
| Available potassium (mg kg^-1^) | 125.2 |
| Cadmium (mg kg^-1^) | 2.1 |
